# Interspecies scaling of suprachoroidal drug delivery using ocular geometry and drug physicochemical properties

**DOI:** 10.1038/s41598-025-34631-x

**Published:** 2026-01-16

**Authors:** Madhoosudan A. Patil, Brock A. Matter, Cleildo Santana, Uday B. Kompella

**Affiliations:** 1 Department of Pharmaceutical Sciences, University of Colorado Anschutz Medical Campus, 12850 East Montview Boulevard, C238-V20, Aurora, CO USA; 2 Department of Ophthalmology, University of Colorado Anschutz Medical Campus, 12850 East Montview Boulevard, C238-V20, Aurora, CO USA; 3 Department of Bioengineering, University of Colorado Anschutz Medical Campus, 12850 East Montview Boulevard, C238-V20, Aurora, CO USA; 4 Colorado Center for Nanomedicine and Nanosafety, University of Colorado Anschutz Medical Campus, 12850 East Montview Boulevard, C238-V20, Aurora, CO USA

**Keywords:** Suprachoroidal delivery, Drug cassette, Ocular drug delivery, Corticosteroids, Beta blockers, NSAIDs, Diseases, Drug discovery, Medical research

## Abstract

Excised rabbit, porcine, and bovine eyes were injected in the suprachoroidal space with a cassette of 27 drugs, including 10 beta-blockers, 8 NSAIDs, and 9 corticosteroids, spanning a lipophilicity range (LogD_7.4_: −4.18 to 4.70). Drug concentrations in retina and vitreous humor were quantified by LC-MS/MS on both injected and non-injected sides. Most drugs were detected in both tissues, with higher concentrations on the injected side (0.04–3.69 fraction dose or FD/g) compared to the non-injected side (0.00–0.74 FD/g), after excluding eight outliers (drug–prodrug pairs and unstable drugs). Total retinal concentrations (0.07–1.88 FD/g) exceeded vitreous concentrations (0.02–0.40 FD/g). Across species, delivery decreased with eye diameter (rabbit > porcine > bovine). For individual species, multiple linear regression explained 70–83% of variance in retinal concentrations and 45–62% in vitreous concentrations using drug physicochemical properties. A unified equation incorporating 1/d² (d is eye diameter in cm), lipophilicity (LogD_7.4_), molar solubility (LogS), and total polar surface area (TPSA) predicted concentrations in all species for a given tissue, explaining 74% of retinal and 93% of vitreous variance. Retinal delivery scaled for all species: Log FD/g = -1.121 + 0.255*LogD_7.4_ + 0.106*LogS + 0.004*TPSA + 0.395*1/d^2^. Vitreous humor delivery scaled for all species: Log FD/g = -1.898–0.047*LogD_7.4_ − 0.051*LogS + 0.001*TPSA + 0.833*1/d^2^. While this study evaluated static barriers, in vivo delivery is expected to be lower due to dynamic clearance mechanisms.

**Supplementary Information:**

The online version contains supplementary material available at 10.1038/s41598-025-34631-x.

## Introduction

Suprachoroidal drug delivery is a new approach to treat eye diseases. The therapeutic benefit of this approach was validated with the approval of the first suprachoroidally injected suspension of a corticosteroid, triamcinolone acetonide (XIPERE™, Clearside Biomedical, Atlanta, GA), by the US FDA in 2021. Suprachoroidally injected triamcinolone acetonide (TA) suspension is useful in treating macular edema and improving vision in patients with uveitis^1^.

A variety of drug molecules beyond corticosteroids including tyrosine kinase inhibitors, anti-VEGF antibodies, complement factors, and gene therapies may be administered by the suprachoroidal route for treating eye diseases in future^2^. For treating back of the eye diseases, two key targeted tissues for drug delivery are the retina and the vitreous humor. However, for suprachoroidal drug delivery to the retina and vitreous humor, the influence of drug properties and species differences are not well understood. In the past, with the objective of understanding small molecule delivery, we determined the permeability of mannitol, fluorescein, budesonide, celecoxib, and rhodamine-6G as marker solutes across isolated bovine and porcine sclera-choroid layers^3^. However, there is no comprehensive study assessing the influence of drug properties on suprachoroidal delivery.

Human pharmacokinetics are rarely feasible or performed since multiple invasive eye samplings can be injurious. Therefore, drug delivery assessment for the eye is largely limited to animal models like rabbits and pigs, which were employed in this study. Species similarities across pigmented and nonpigmented eyes can be reassuring in scaling the data to humans. In this study, porcine and bovine eyes were pigmented, while rabbit eyes were nonpigmented. A traditional approach is to use drug delivery in rabbit to predict another model and if the prediction is accurate, then the model is applied to the human eye. Thus, understanding the influence of drug properties on tissue distribution is very important for early candidate selection and formulation development. Finally, ex vivo studies, as performed in this study, favor high throughput screening of drug candidates, while obeying 3R principles^4^.

Additionally, the traditional approach to drug delivery is to evaluate one drug at a time. To increase the throughput, N-in-one dosing, or a cassette dosing approach was used in this study to elucidate the influence of small molecule drug properties on suprachoroidal drug delivery. Prior to this work, the lab used this drug cassette technique in other studies, including (1) a cassette containing less than ten drugs from several different drug classes which evaluated the influence of drug physicochemical properties on delivery to brain mitochondria^5^, and (2) the influence of drug physicochemical properties on rabbit, porcine, and bovine corneal delivery of twenty-five different drugs in a cassette dosing approach^6^. For this study, a larger cassette of twenty-seven drugs from three different drug classes, beta-blockers, NSAIDs, and corticosteroids, was dosed using a suprachoroidal injection in isolated rabbit, porcine, and bovine eyes and drug delivery to the retina and vitreous humor was assessed. The drug molecules used in this study have positive, negative, or neutral charges at physiological pH. Drug delivery to the retina and vitreous humor was further predicted based on the eye diameter as well as drug physicochemical properties.

## Materials

### Drug cassette and chemicals

The constituents of the 27-drug loaded cassette were selected from three different drug classes, i.e., 10 beta-blockers, 8 NSAIDs, and 9 corticosteroids. The beta-blockers atenolol, sotalol hydrochloride, nadolol, timolol maleate, pindolol, metoprolol tartrate, alprenolol hydrochloride, betaxolol hydrochloride, and propranolol hydrochloride were purchased from Sigma-Aldrich (St. Louis, MO, USA). The remaining drug from the beta-blocker class, oxprenolol hydrochloride, was purchased from MP Biomedicals (Santa Ana, CA, USA). The NSAIDs tolmetin sodium dihydrate, ketoprofen, indoprofen, bromfenac sodium, naproxen, nepafenac, mefenamic acid, and flupirtine maleate were purchased from Sigma-Aldrich. The corticosteroids dexamethasone disodium phosphate, triamcinolone, prednisolone, and prednisolone 21-acetate were purchased from Sigma-Aldrich. The corticosteroids, fluocinolone acetonide, budesonide, difluprednate, and triamcinolone hexacetonide were purchased from Spectrum Chemical (New Brunswick, NJ, USA). The corticosteroid dexamethasone was procured from Sigma-Aldrich (St. Louis, MO; purity ≥ 98%) or Shanxi Jinjin Chemical Company Ltd. (Hejin, Shanxi, China; purity ≥ 97%). Internal standards used for the LC/MS analysis were Dexamethasone-4,6α,21,21-d4 purchased from CDN Isotopes, Quebec, Canada, Flupirtine-d4 hydrochloride, and timolol-d5 maleate purchased from Santa Cruz Biotechnology, Santa Cruz, CA, USA. Additional chemicals, calcium chloride, dimethyl sulfoxide (DMSO), formic acid (CH_2_O_2_), magnesium chloride heptahydrate, magnesium sulfate heptahydrate, methanol, and sodium fluorescein (NaF) were purchased from Sigma-Aldrich, St. Louis, MO, USA. Acetonitrile, potassium chloride, potassium phosphate dibasic, sodium chloride, and sodium phosphate dibasic were purchased from Fisher Scientific, Pittsburgh, PA, USA. Table 1 lists the 27 drugs, internal standards, drug class, molecular weights, and predicted LogD_7.4_ values^6,7^.

**Table 1:** List of drugs and internal standards in the drug cassette, drug class, molecular weights, and predicted LogD_7.4_ values.

Name of drug with salt form	Drug	Mol wt	Predicted
(In order of LogD_7.4_ values)	Class	(g/mol)	LogD_7.4_
Atenolol	Beta-blocker	266.34	−1.85
Sotalol HCl	Beta-blocker	308.82	−1.54
Nadolol	Beta-blocker	309.40	−0.86
Timolol maleate	Beta-blocker	432.49	−0.34
Pindolol	Beta-blocker	248.32	−0.32
Metoprolol tartrate	Beta-blocker	684.81	−0.29
Oxprenolol HCl	Beta-blocker	301.81	0.18
Alprenolol HCl	Beta-blocker	285.81	0.68
Betaxolol HCl	Beta-blocker	343.89	0.77
Propranolol HCl	Beta-blocker	295.80	1.15
Tolmetin sodium dihydrate	NSAID	315.30	−0.67
Ketoprofen	NSAID	254.28	0.05
Indoprofen	NSAID	281.31	0.14
Bromfenac sodium	NSAID	356.15	0.2
Naproxen	NSAID	230.26	0.45
Nepafenac	NSAID	254.28	1.38
Mefenamic acid	NSAID	241.29	2.04
Flupirtine maleate	NSAID	420.39	2.47
Dexamethasone sodium phosphate	Steroid	516.40	−4.18
Triamcinolone	Steroid	394.44	0.92
Prednisolone	Steroid	360.44	1.66
Dexamethasone	Steroid	392.46	1.92
Prednisolone 21-acetate	Steroid	402.50	2.33
Fluocinolone acetonide	Steroid	452.49	2.39
Budesonide	Steroid	430.53	3.02
Difluprednate	Steroid	508.55	3.17
Triamcinolone Hexacetonide	Steroid	532.64	4.70
**Internal standards (ISTD)**			
Timolol-d_5_ maleate	Beta-blocker	437.52	-----
Flupirtine-d_4_ HCl	NSAID	344.81	-----
Dexamethasone-d_4_	Steroid	396.49	-----

## Methods

### Preparation of standard solutions

Stock drug solutions were prepared by separately weighing ≥ 1 mg each drug and internal standard then dissolving each drug in DMSO or water (e.g., bromfenac sodium and tolmetin sodium dihydrate were prepared in water). The stock injection solution was prepared by mixing stock drug solutions together in DMSO to reach a concentration of 200 µg/mL for each drug. The injection solution used for treatment of the eyes was made by mixing 5 µL of the stock injection solution with 995 µL of water to give a solution that contained 1 µg/mL of each drug in 0.5% DMSO in water. Calibration solutions were made by mixing equal molar amounts of each drug into a solution of DMSO then making serial dilutions in DMSO.

### Drug dosing, incubation, and sampling

No live animals were used in this research. No animals were sacrificed specifically for this research. Eyes were obtained post-mortem from commercial abattoirs in compliance with USDA regulations and local ethical standards. Institutional Animal Care and Use Committee approval of a protocol was not required because no live animals were involved. New Zealand white (NZW) albino rabbit eyes were purchased from Pel-Freez (Rogers, AR, USA). Freshly excised rabbit eyes were shipped overnight in Hank’s Balanced Salt Solution (HBSS) buffer on ice. Freshly excised bovine and porcine eyes were obtained from a local abattoir (Elizabeth Locker Plant, Elizabeth, CO, USA). The eyes were immersed in containers with HBSS immediately after being excised and placed on ice by abattoir employees, then picked up, and transported by a laboratory member to the university lab. All eyes were used immediately upon receipt.

Eyelids, conjunctiva, and retrobulbar tissues (except optic nerve head) were removed from all the eyes, leaving the cornea and globe intact. Once the excess tissue was removed, globes were visually inspected to ensure that no damage was caused during the removal process. The intact eyes were then placed in a large, 12 well plate containing PBS (pH 7.4) and allowed to equilibrate to 37 °C over 30 min in a water bath. Subsequently, 25, 50, or 100 µL PBS containing 1 µg/mL of each drug in the cassette was injected using a Hamilton syringe with a 25G 5/8” 45° beveled needle into the suprachoroidal space of the rabbit, porcine, and bovine eyes, respectively. The dosing solution also contained sodium fluorescein dye at 10 µg/mL; this dye was not considered part of the drug cassette. The needle was inserted in a location that was posterior to the limbus at an angle of 45^o^ and the syringe plunger was slowly pushed to inject the solution. After the dose was injected, the needle was held in place for 10–15 s before gently pulling it out of the globe. Using tissue marking dye, a mark was made on the injection side of each eye. The eyes were placed back into the well plate and incubated for one hour. Immediately after the incubation, the eyes were frozen using dry ice and stored in the freezer until further processing.

Prior to tissue isolation, eyes were removed from the freezer and exposed to room temperature (RT, ~ 20–22 °C) for 30 min. Subsequently, eyes were cut in half, isolating the injected side and non-injected side, to prevent cross-contamination a new scalpel blade was used for each cut. Retina and vitreous humor were isolated from both sides of each eye using clean tools. Between each sample, all tools used were washed thoroughly with DMSO: water and blotted dry to prevent cross-contamination between samples. Isolated tissues were placed in pre-weighed Axygen™ plastic tubes (Fisher Scientific, Waltham, MA) then reweighed to obtain the tube and actual tissue weight. Collected tissues were frozen until analysis.

### Extraction of drug cassette

Representative tissue samples were collected from the injected and non-injected side of each. Tissue samples were mixed with phosphate-buffered saline (PBS), at pH 7.4, spiked with the internal standard mix, then subjected to three freeze-thaw cycles (using liquid nitrogen to freeze than warming to room temperature) to homogenize the tissues. Although, vitreous humor samples did not require homogenization, they were also subjected to the freeze-thaw procedure to ensure sample handling was as consistent as possible between sample types. Following homogenization, samples were mixed with 3x their volume of 2:1 acetonitrile: methanol. Samples were vortexed, then incubated for 30 min at room temperature. Following incubation, samples were mixed for 10 min using a multi-tube vortexer (Model VX-2500 from VWR, Radnor, PA), bath sonicated for 5 min, then placed on ice to cool. The collected samples were centrifuged at 13,000 rcf for 10 min at RT. The supernatants from each sample were collected into a fresh Axygen™ plastic tube without disturbing the pellet. Samples were concentrated using a Savant™ SC100 SpeedVac (Holbrook, NY, USA which is now part of thermo Fisher Scientific, Waltham, MA, USA), until the volume remaining was around 100 µL. Samples were frozen pending LC-MS/MS analysis.

### LC-MS/MS analysis

LC-MS/MS analysis was performed as per our lab’s method described in Matter, B. et al., 2022^7^, which is further elaborated below.

### Instrumentation

A Prominence HPLC (Shimadzu, Kyoto, Japan) coupled to a QTrap 4500 mass spectrometer (AB Sciex, Farmingham, MA) was used for this analysis. The mass spectrometer was operated in the ESI + mode^7^. All the settings were optimized by manually tuning to infused standard concentration solutions. Global mass spectrometer parameter settings were selected to give the highest average sensitivity for all drugs in the cassette. Global mass spectrometer settings are as follows: curtain gas, 45 psi; collision gas, high; ion spray voltage, 5000 V; source temperature, 500 °C; ion source gas 1 and 2, 50 psi; entrance potential, 10 V; and collision cell exit potential, 14 V^7^. For the specific transitions monitored in the mass spectrometer, optimized declustering potential (DP; volts), and collision energies (CE; volts) which were optimized separately for each drug^7^. The data was collected using scheduled multiple reaction mode (MRM) with a detection window of ± 45 s and a cycle time of 0.2 s^7^. Quantitation was performed using stable isotope dilution with dexamethasone-d_4_, flupirtine-d_4_, and timolol-d_5_ added as internal standards for corticosteroids, NSAIDs, and beta blockers, respectively. The samples with drug levels below the quantitation limit were considered zero^7^.

### Chromatographic conditions

Analytical separation was achieved with a Phenomenex kinetex C18 column [2.1 × 100 mm, 2.6 μm]^7^. The column was held at 40 °C and eluted at 0.6 mL/min with a gradient of 0.1% formic acid (A) and 0.1% formic acid in 9:1 acetonitrile: water (B) with a total runtime of 18 min. Chromatographic separation was achieved with a linear gradient (time, % of solvent B): 0–2 min, 0.6% B; 2–2.5 min, 0.6–6.6% B; 2.5–10.5 min, 6–53% B; 10.5–14 min, 53–94% B; 14–14.5 min, 94 − 0.6% B; and then isocratic for 3.5 min at 0.6% B to re-equilibrate the column^7^. The first 3 min of the eluant was diverted to waste to reduce front end contamination of the mass spectrometer^7^.

### Multiple linear regression (MLR) modelling

Multiple linear regression was performed as per our previously described approach^6^, which is further elaborated below.

LC/MS quantification data was used to obtain predictive linear models of drug uptake for each tissue. Multiple linear regressions were performed using the least squares method with Microsoft Excel^®^ Professional 2016^6^. The following molecular descriptors were calculated for each drug with ACDLabs^®^, version 2019, and used as independent variables: molecular weight, number of hydrogen bond donors, number of hydrogen bond acceptors, number of hydrogen bond donors and acceptors, total polar surface area (TPSA), number of rotatable bonds, carbon ratio, nitrogen ratio, nitric oxide ratio, heteroatom ratio, halogen ratio, number of rings, number of aromatic rings, number of 5 atom rings, number of non-aromatic 6 atom rings, Log(bioconcentration factor or BCF), parachor, index of refraction, surface tension, density, polarizability, molar volume, molar refractivity, LogS, LogP, LogD at pH of 7.2, 7.3, 7.4, 7.5, and 7.6^6^.

**Fig. 1:**
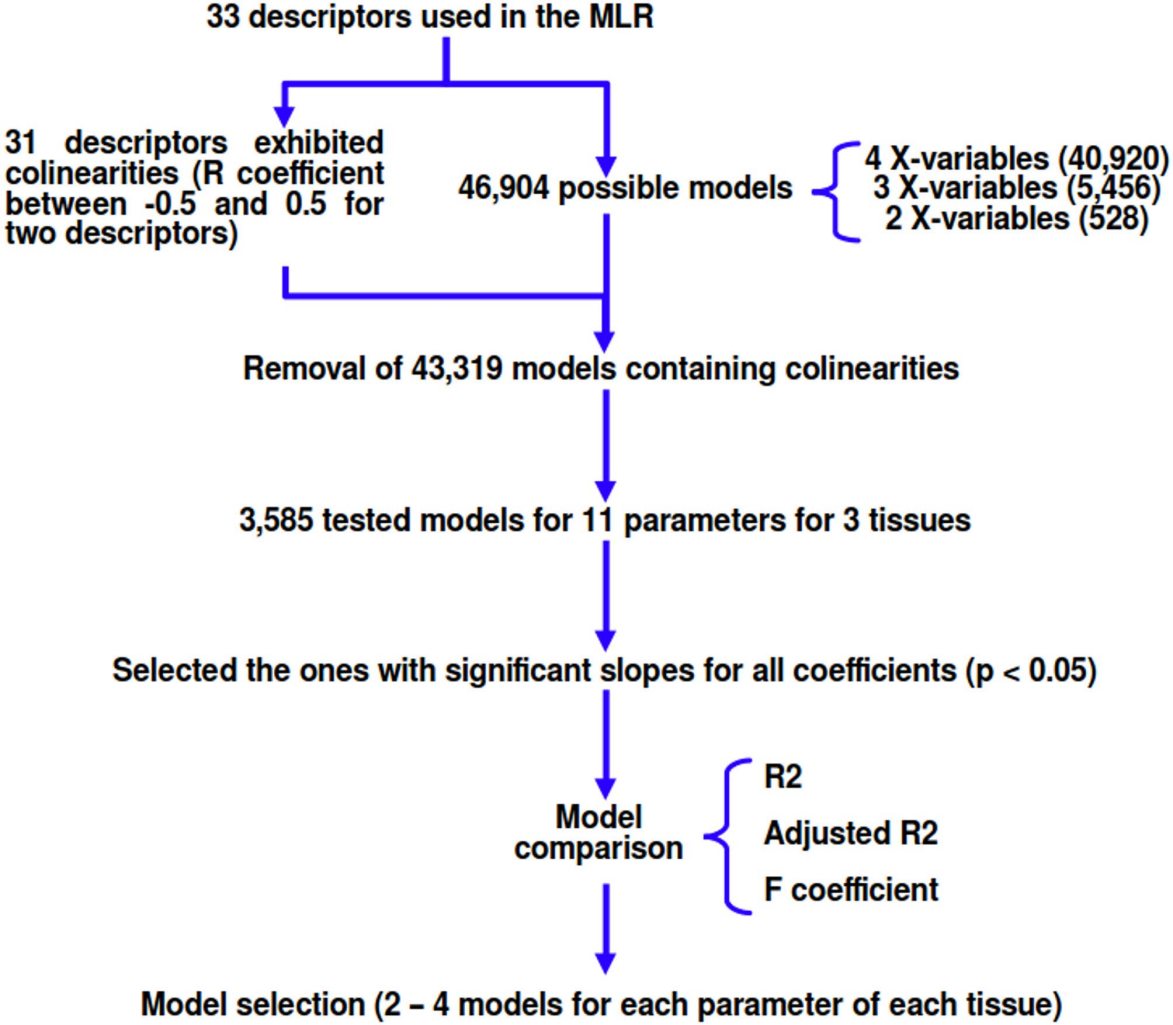
Flowchart for multiple linear regression. This rationale was used for obtaining MLR models and evaluation of the models obtained.

To select best-fit models, all possible collinearity-free models with four, three and two independent variables were obtained^6^, as described in Fig. 1. Only the models presenting significant (*p* < 0.05) coefficients for all independent variables were selected and evaluated by R^2^, adjusted R^2^, and F values. Additionally, combining data for all species, species-specific 1/d^2^ (d = eye diameter in cm) parameter was incorporated into MLR models (R version 4.5.2) along with commonly used drug physicochemical properties including LogD_7.4_, molar solubility (LogS), and total polar surface area (TPSA) to predict tissue concentrations.

## Results

### LC-MS analysis

The lower limits of quantitation (LLOQ) were determined for each of the 27 cassette drugs by injecting drug cassette standard sets starting at 50 attomoles on column into the LC-MS/MS^7^. This concentration was increased with each new set of injections until an accuracy of between 80 and 120% was obtained with a precision of 20%. Drug LLOQ values ranged from 0.5 to 40 femtomoles. Instrument sensitivity correlated with ionization and fragmentation efficiency. Interestingly, certain trends were recognized for specific drug characteristics. For instance, small drug molecules (e.g., sotalol) had greater sensitivity due to fewer possible MS/MS transitions (500 attomoles on column). Any drugs (e.g., ketoprofen) with poor ionization efficiency in positive mode or many MS/MS transitions (e.g., dexamethasone) had values at 40 femtomoles on column. For those drugs with bromine present like bromfenac the signal was split by the two isotopes, which also resulted in values of 40 femtomoles on column. As for the drug budesonide with two structural isomers, which split the peak, the total signal intensity resulted in an LLOQ of 25 femtomoles on column.

Accuracy and precision of the mass spectrometer for intra-day and inter-day weighted calibration curves for the drugs over the entire concentration range gave the most consistent results. Most drugs had an accuracy of 85–115% and precision < 15%. The early eluting drugs were sensitive to minor differences in solvent composition that resulted in variances at the low end of the calibration curve (e.g., sotalol, atenolol, and pindolol), resulting in reduced accuracy and precision at the ≤ 40 femtomole concentration. One of the drugs, triamcinolone hexacetonide, showed greater than desired variances at all calibration curve concentrations.

### Data outliers

Based on the high levels of flupirtine delivery and further analysis, we learned that there was an interference in its quantification. Therefore, it was omitted from any correlations. Additionally, drug-prodrug pairs were omitted from correlations since prodrug to drug conversion confounds the quantification of the drug delivered. Due to variability in tissue analysis of difluprednate, triamcinolone hexacetonide, dexamethasone, its prodrug dexamethasone sodium phosphate, prednisone, and its prodrug prednisolone 21-acetate they were omitted from the correlations. The figure legends indicate the omission of outliers, where appropriate.

### Drug delivery to the injected and/or non-injected side

**Fig. 2:**
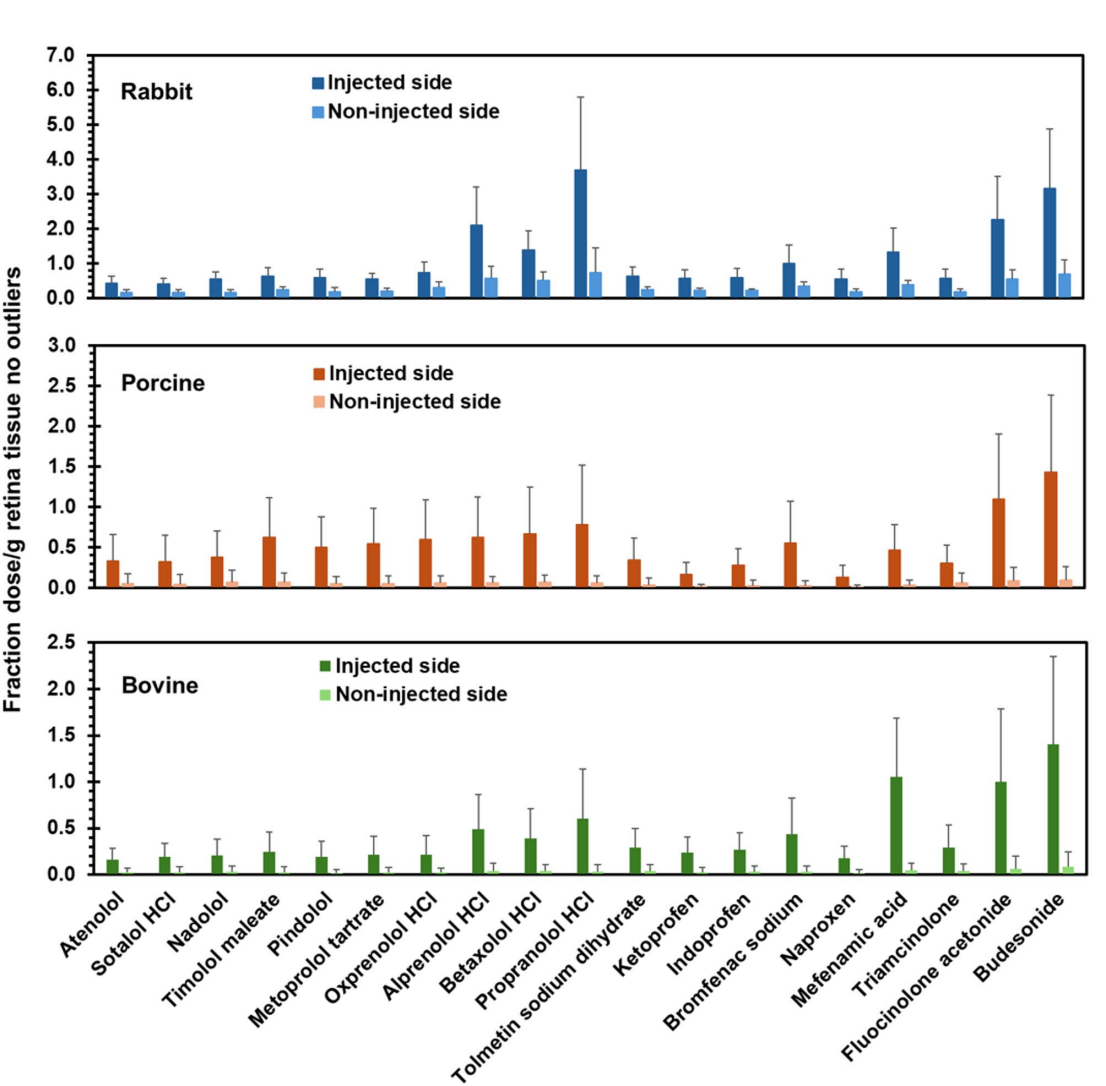
Retinal drug delivery to the injected and non-injected side of eyes from three species at 1-hour following suprachoroidal injection. The data is presented as mean ± STDEV for *n* = 6 eyes; outliers were omitted.

**Fig. 3:**
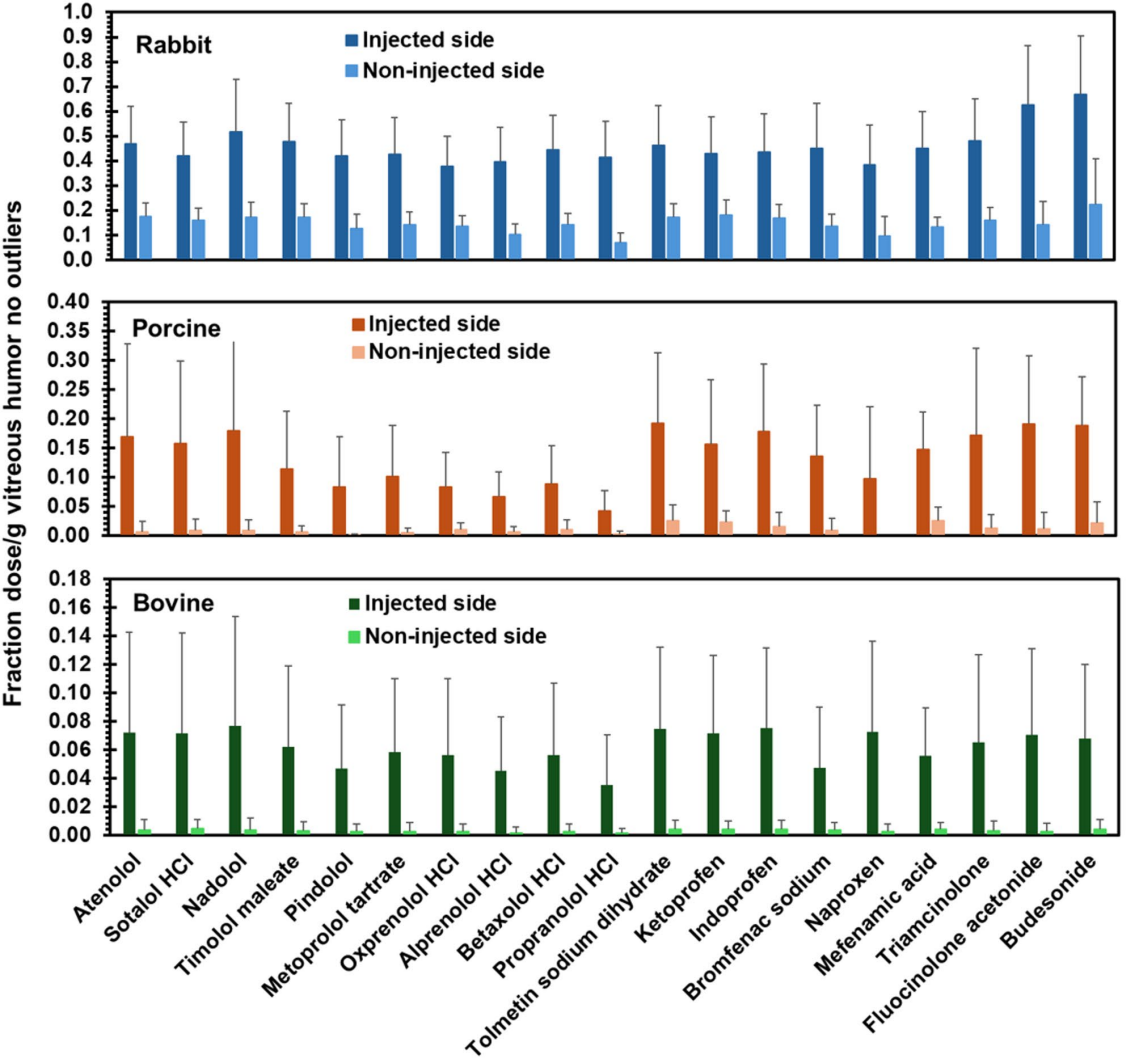
Vitreous humor drug delivery to the injected and non-injected side of eyes from three species at 1-hour following a suprachoroidal injection. The data is presented as mean ± STDEV for *n* = 6 eyes; outliers were omitted.

**Fig. 4:**
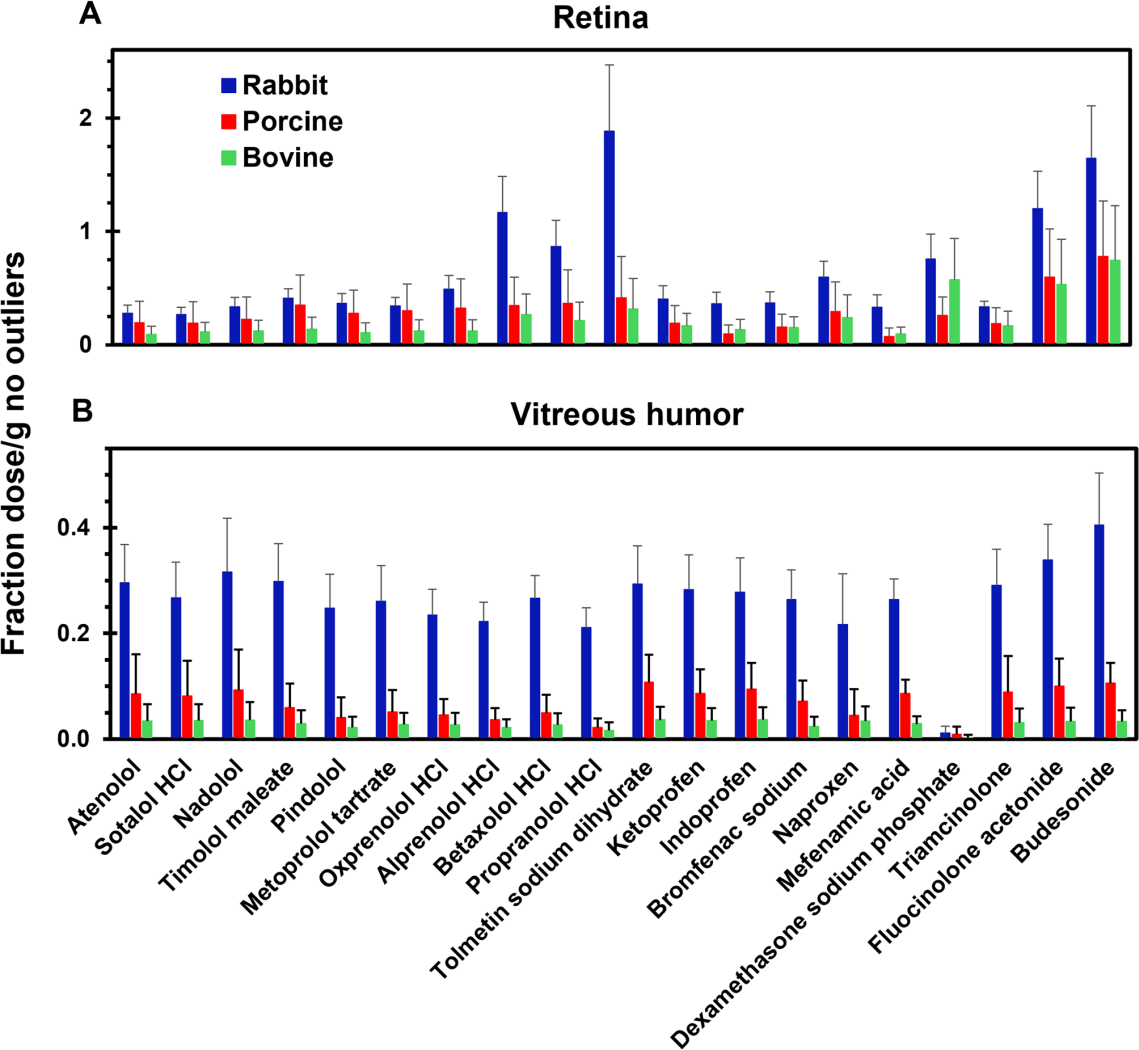
**A**, **B** Total retinal and total vitreous humor drug delivery to the injected and non-injected side in the eyes of three species at 1-hour following a suprachoroidal injection. The data is presented as mean ± STDEV for *n* = 6 eyes; outliers were omitted.

Because actual doses varied from theoretical amounts, data is shown as fraction of dose per gram of tissue in all figures. Data is plotted for rabbit, porcine, and bovine eyes in each figure, unless otherwise indicated. Figures 2 and 3 show drug levels on the injected side and non-injected side in retina and vitreous humor with no outliers, respectively. The supplemental Figs. 1 and 2 just show the outliers. In all three species, drug delivery to the injected side of the retina and vitreous humor was higher compared to the non-injected side for almost all of the drugs. Since each half of the eye may not be exact in dissection and quantification, total drug delivered to the retina and vitreous humor combining the injected and non-injected side is also shown in Fig. 4 for retina and vitreous humor, respectively. The supplemental Fig. 3 shows just this data for outliers. Based on this data, total drug delivery to the retina and vitreous humor was generally higher in rabbits compared to the porcine and bovine eyes for all classes of drugs. Supplemental Fig. 4 shows the heatmap of Pearson’s correlation coefficients for inter-species correlations for vitreal and retinal concentrations after omitting outliers. Interestingly, there is good correlation between the species for both tissues in this regard and rabbit retinal delivery correlated well with porcine retina.

### Correlation of drug delivery to Lipophilicity, total Polar surface Area, and solubility

**Fig. 5:**
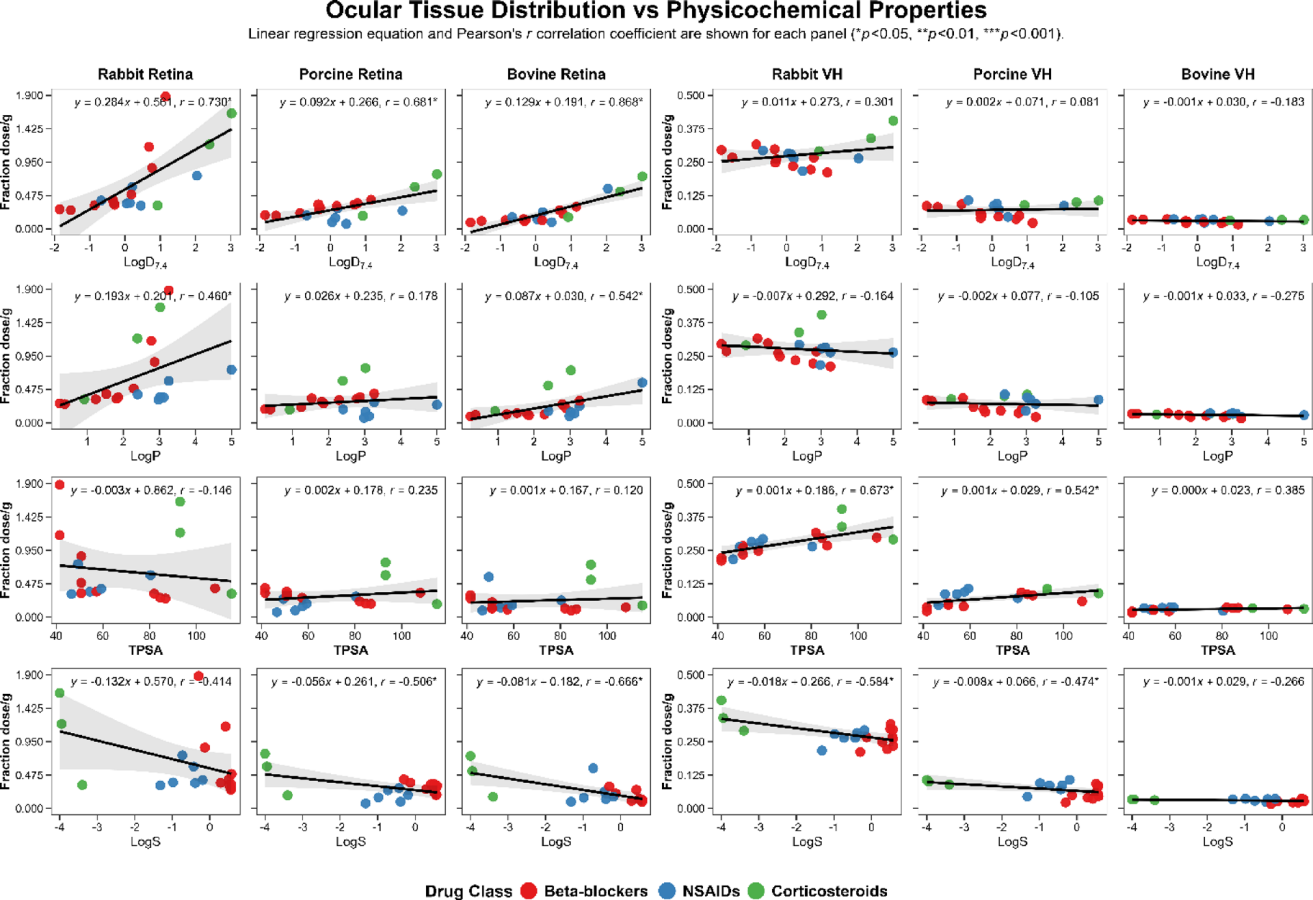
Correlation of suprachoroidal drug delivery to the retina or vitreous humor with common drug physicochemical properties, including lipophilicity (Log D_7.4_ and Log P), total polar surface area (TPSA, Å^2^), and molar solubility (Log S) in three species. In general, Pearson’s correlation coefficient was the highest for Log D_7.4_ followed by Log S for predicting retinal concentration. Vitreous drug concentrations correlated the best with TPSA followed by LogS. Injected drugs from all three drug classes, beta-blockers, NSAIDs, and corticosteroids, were used. Total drug concentration combining injected and non-injected side was plotted. Outliers were omitted.

Figure 5 shows Pearson’s correlation coefficients for retinal or vitreal drug delivery for all the drug classes combined and LogD_7.4_, LogP, TPSA, and molar solubility (LogS). It is evident that LogD_7.4_ predicts retinal delivery better than LogP in all three species, with the delivery increasing with an increase in LogD_7.4_. Further, it is the best parameter among the above four to predict retinal delivery. On the other hand, vitreal delivery was best predicted by TPSA, with the delivery increasing with an increase in TPSA in rabbit and porcine eyes. However, these trends differed when drug molecules were considered by drug class.

Since lipophilicity is a key physicochemical property influencing drug delivery across biological membranes, supplemental Fig. 5 shows these correlations by drug classes for beta-blockers, NSAIDs, and corticosteroids. In Fig. 5, outliers were omitted, which included 2 NSAIDS and 6 corticosteroids as referred to below. Drug delivery to the retina generally showed a positive correlation with LogD_7.4_ for all three drug classes individually or when combined. For the combined data, the coefficient determination was fair and ranged from 0.46 to 0.68 for the retina. For individual drug classes the correlations were fair, except for NSAIDs in porcine retina, where the correlation was poor with a coefficient of determination of only 0.10. For the combined data for all drugs in vitreous humor, the coefficient of determination was poor and ranged from 0.01 to 0.10. For vitreous humor delivery, the correlations were fair to good for beta-blockers (coefficient of determination: 0.49 to 0.76) and poor for NSAIDs (coefficient of determination: 0.06 to 0.18), even after omitting nepafenac and flupirtine maleate, and they tended to omit "continue to" decline with lipophilicity. For the corticosteroids, there was a good positive correlation (R^2^ = 0.90 to 1.0), after omitting the outliers with low reliability in measurement including some drug-prodrug combinations (e.g., dexamethasone, its prodrug dexamethasone sodium phosphate, difluprednate, prednisone, its prodrug prednisolone 21-acetate, and triamcinolone hexacetonide).

### Correlation of drug delivery with eye diameter

**Fig. 6:**
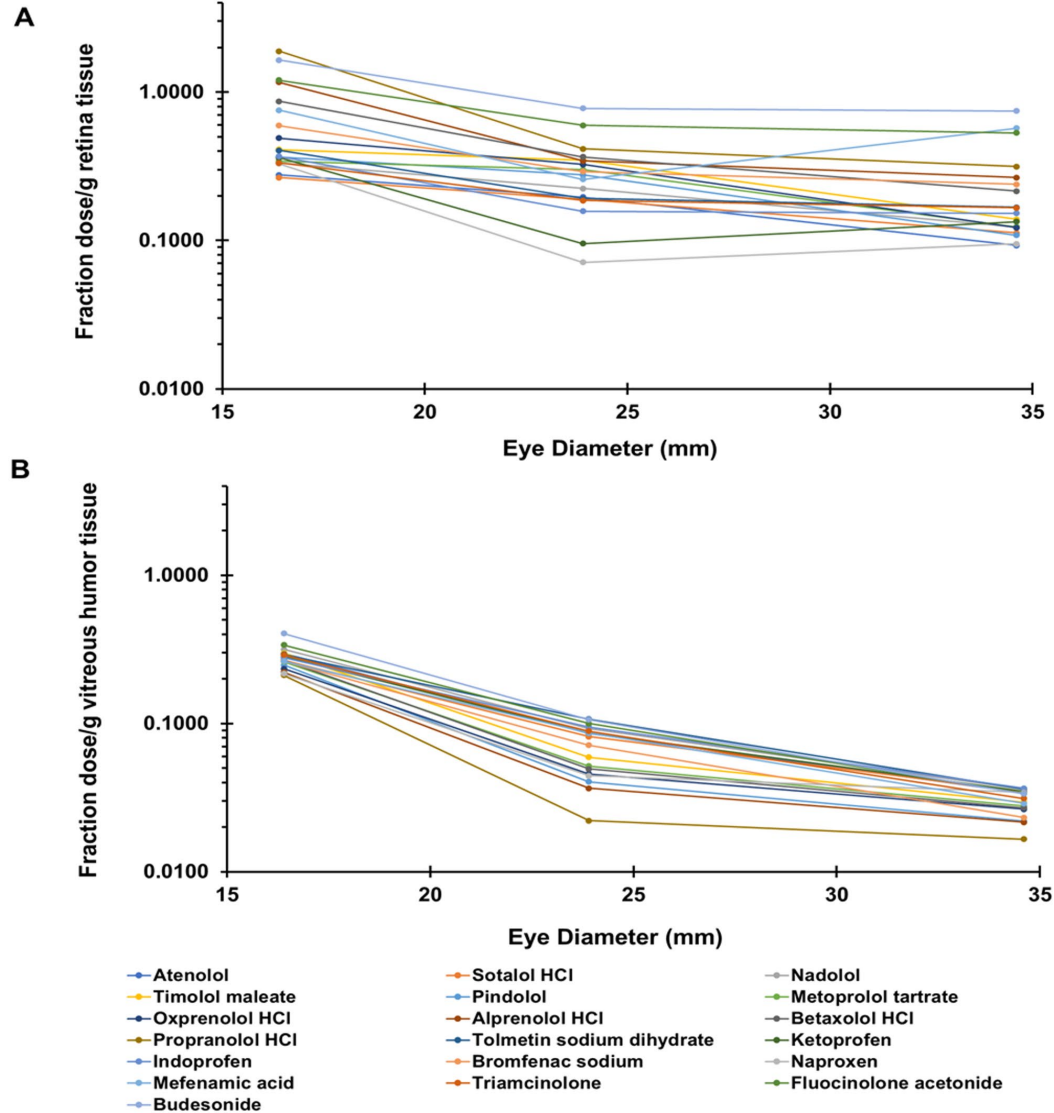
**A**, **B** The effect of eye diameter on suprachoroidal drug delivery. The average fractions obtained for each tissue, A - retina and B - vitreous humor, were plotted against eye diameter for rabbit, porcine, and bovine eyes. Total drug concentration combining injected and non-injected side is plotted. Outliers were omitted.

Figure 6A and B correlates drug delivery to eye diameter for various drugs. Average eye diameters reported in literature were used (i.e., rabbit 16.4, porcine 23.9, and bovine 34.6 mm), since each eye used in the study was not physically measured for its diameter^8–11^.

Retinal and vitreous humor delivery of prodrugs appeared to be extremely low compared to its corresponding drug, indicating the conversion of prodrug to drug during the experiment (Supplemental Fig. 3).

### MLR modeling

Best-fit models obtained were ranked considering adjusted R^2^ and the models with best performance for each tissue are described in Table 2.

**Table 2:** Best-fit predictive models (intercept + x-variables) were obtained for retina and vitreous humor in three different species (rabbit, porcine, and bovine). The best equation based on adjusted R^2^ is shown at the top of each category. Additionally, the next best equations incorporating LogD_7.4_, LogP, or TPSA are also shown. All equations shown are statistically significant correlations.

**Tissue**	**Species**	**Model**	**R** ^2^	**Adjusted R** ^2^	**F**
Retina	Bovine	– log FD g-1 = (7.93527) + (0.0416 RB) – (1.8073 NR) – (0.2317 LBCF) – (2.9375 LMV)	0.850	0.808	19.90
		– log FD g-1 = (2.21001) – (0.0351 POL) – (0.1512 LP)	0.800	0.775	32.04
		– log FD g-1 = (1.67045) – (0.0077 TPSA) – (0.2916 LBCF)	0.667	0.625	16.03
	Porcine	– log FD g-1 = (8.58259) – (3.4695 LMW) – (2.9869 NR) – (0.1483 LBCF) + (0.0192 ST)	0.865	0.827	22.49
		– log FD g-1 = (5.29441) – (2.1535 LMW) – (2.8458 NR) + (0.0164 ST) – (0.1185 LD_7.4_)	0.841	0.796	18.55
		– log FD g-1 = (8.4088) – (3.4025 LMW) – (2.6633 NR) + (0.0198 ST) – (0.1079 LP)	0.833	0.785	17.41
		– log FD g-1 = (1.2585) – (0.0043 TPSA) – (0.0568 RB) – (0.1577 LD_7.6_)	0.689	0.627	11.10
	Rabbit	– log FD g-1 = (0.51743) – (0.0316 RB) – (0.1995 LogD_7.5_)	0.733	0.700	21.99
		– log FD g-1 = (0.53738) – (0.036 RB) – (0.1996 LD_7.4_)	0.729	0.695	21.49
		– log FD g-1 = (1.18261) + (0.0118 ST) – (0.0142 MR) – (0.1422 LP)	0.734	0.681	13.80
		– log FD g-1 = (1.09806) – (0.1668 LBCF) + (0.0106 ST) – (0.0135 MR)	0.704	0.644	11.88
Vitreous humor	Bovine	– log FD g-1 = (3.54389) + (0.0724 HBD) + (0.1478 AR) – (1.5133 IR) + (0.0748 LD_7.6_)	0.631	0.525	5.97
		– log FD g-1 = (3.86732) + (0.0791 HBD) + (0.1511 AR) – (1.7284 IR) + (0.0759 LD_7.4_)	0.607	0.495	5.41
		– log FD g-1 = (1.56701) + (0.0503 HBD) – (0.0032 TPSA) + (0.9424 NR) + (0.0472 LD_7.6_)	0.572	0.450	4.68
	Porcine	– log FD g-1 = (1.57695) + (0.0454 RB) – (0.001 PA) + (0.0947 LD_7.6_)	0.543	0.451	5.94
		– log FD g-1 = (1.51844) + (0.0485 RB) – (0.001 PA) + (0.0872 LD_7.4_)	0.515	0.418	5.32
	Rabbit	– log FD g-1 = (0.87896) + (0.0088 RB) – (0.0006 PA) + (0.0247 LD_7.6_)	0.687	0.624	10.96
		– log FD g-1 = (0.86457) + (0.0097 RB) – (0.0006 PA) + (0.0231 LD_7.4_)	0.675	0.610	10.40

FD: Fraction of Dose; LMW: Log Molecular Weight; LMV: Log Molar Volume; HBD: Hydrogen Bond Donors; RB: Rotatable Bonds; HBA: Hydrogen Bond Acceptors; NR: N Ratio; LP: LogP; HR: Hetero Ratio; LBCF: Log(BCF): logarithm of the bioconcentration factor ; LogS: Log Molar Solubility; ST: Surface Tension; AR: Aromatic Rings; LD: LogD_7.4_ or LogD_7.5_, or LogD_7.6_; MV: Molar Volume; IR: Index of Refraction; MR: Molar Refractivity; PA: Parachor; POL: Polarizability; and TPSA: Total Polar Surface Area.

Further MLR analysis incorporating 1/d^2^ parameter indicated the following two statistically significant correlations for predicting retinal and vitreous humor concentrations in any species.

*Retinal delivery scaled for all species (R*^2^ = *0.759 and adjusted R*^2^ = 0.741): log FD g^− 1^ = −1.121 + 0.255*LogD_7.4_ + 0.106*LogS + 0.004*TPSA + 0.395*1/d^2^.

*Vitreous humor delivery scaled for all species (R*^2^ = *0.938 and adjusted R*^2^ = 0.933): log FD g^− 1^ = −1.898–0.047*LogD_7.4_ − 0.051*LogS + 0.001*TPSA + 0.833*1/d^2^.

## Discussion

Suprachoroidal delivery is approved by the US FDA for triamcinolone acetonide, a corticosteroid useful in treating macular edema associated with uveitis. The suspension nature of this product sustains drug delivery for prolonged periods in the back of the eye tissues^12,13^. Alternative strategies include advanced drug delivery systems such as liposomes^12^ and polymeric nanoparticles, microparticles, implants, and in situ formed gels^14–17^. Irrespective of the dosage form used, the drug released in the suprachoroidal space should traverse choroid-RPE to access the retina and vitreous humor in treating macular edema, uveitis, and other related eye pathologies. The entry of drug into the retina and vitreous humor is expected to depend on drug molecular properties including lipophilicity and a multitude of physiological factors such as eye diameter that are species dependent.

This study evaluated the suprachoroidal delivery of 27 drugs belonging to three drug classes, i.e., beta-blockers, NSAIDs, and corticosteroids, to the back of the eye in three species (rabbit, porcine, and bovine) using an ex vivo eye model and correlated drug delivery to the eye diameter and drug lipophilicity. The results generally indicated a decrease in drug delivery with increasing eye diameter and an increase in retinal delivery with higher lipophilicity for all three drug classes. In contrast, the influence of lipophilicity on vitreal delivery varied by drug class, showing a negative correlation for beta-blockers and NSAIDs and a positive correlation for corticosteroids (Supplemental Fig. 5).

We used a validated high throughput LC-MS/MS method for a cassette of twenty-seven drugs, with limits of quantitation in the 0.5 to 40 femtomole range^7^. This sensitive method allowed microgram-level dose of each drug in a cassette (N-in-one) and quantification of the small quantities of multiple drugs in eye tissues. A common approach to drug analysis in eye tissues is to quantify the drug in the entire eye tissue (e.g., in the entire retina or vitreous humor). In this study, drug levels in the retina and vitreous humor were quantified on the injected side as well as the non-injected side by dissecting the eye into two parts (Figs. 2 and 3; Supplemental Figs. 1 and 2). Further, the data is also presented as the sum of the drug amount on both sides or the entire tissue (Fig. 4 and Supplemental Fig. 3). Majority of the drugs were detected in both tissues, with the concentration in the retina and vitreous humor being higher on the injected side (0.035–3.686 fraction dose or FD/g tissue) compared to the non-injected side (0–0.737 FD/g tissue), after excluding outliers including prodrug-drug pairs and drugs that are unstable (Figs. 2 and 3). Concentration in the retina (0.07–1.88 FD/g tissue) was higher than that of vitreous humor (0.02–0.40 FD/g tissue) (Fig. 4). In addition, for most drugs, the total tissue delivery as well as the fraction of drug transported from the injected to the non-injected side in both tissues is in the order rabbit > porcine > bovine, decreasing with an increase in eye diameter (Fig. 6).

Further, drug levels in the retina and vitreous humor for 19 drugs were correlated with several physicochemical properties using multiple linear regression to arrive at the best equations to predict drug delivery (Table 2). In general, MLR based on drug properties predicted retinal delivery better than vitreous humor delivery, with 70–83% and 45–62% variance explained by the equations for retina and vitreous, respectively. Log(BCF) or the logarithm of the bioconcentration factor was present in the most predictive equations for retinal delivery but not for vitreal delivery. Log(BCF) is the ratio between the concentration of a substance in an aqueous environment and the concentration of the same substance in an aquatic organism, whose exposure is not through diet. It is a coefficient that more directly measures the tendency of a molecule to concentrate in a biological system, and it has a high correlation with LogP^18^. Although this descriptor is more commonly used for toxicological studies, it is relevant for relating the tendency of a drug to accumulate in a tissue. Additionally, drug delivery comparison with commonly used physicochemical properties in quantitative structure activity relationship studies indicated that retinal delivery increases with LogD_7.4_ (Fig. 5). Vitreal delivery on the other hand increases with TPSA.

Retinal drug concentrations increased with lipophilicity in all three species for the three classes of drugs, with the correlation for increase being poor for NSAIDs in porcine eye (R^2^ = 0.1 vs. 0.4–1.0.4.0 for beta-blockers and corticosteroids) (Supplemental Fig. 5). The reasons for low correlation in porcine eye are unclear and need to be explored further in future studies. Vitreous humor concentrations decreased with lipophilicity in all three species for beta-blockers (R^2^ = 0.5–0.8) and increased for corticosteroids (R^2^ = 0.9–1.0.9.0). A negative trend was also observed for vitreal delivery of NSAIDs, with a low correlation coefficient. Prodrug to drug conversion was evident for dexamethasone phosphate, prednisolone-21-acetate, and nepafenac, with nearly complete conversion in all three species for dexamethasone phosphate and prednisolone-21-acetate (Supplemental Fig. 3).

Good correlations were observed for drug concentrations between the same tissues of different species (Supplemental Fig. 4). Although tissue levels correlated well between species, rabbit tissue data exhibited wider confidence intervals in the correlation plots, indicating greater predictive variability for rabbit data (Fig. 5). The correlations between vitreous humor and retina concentrations were weak, suggesting that different physicochemical properties contribute to vitreal vs. retinal drug delivery. Interestingly, the correlation between vitreous humor and retina, albeit weak, was higher for rabbit, compared to the pigmented species. Further, when related to eye diameter, with an increase in diameter, the decline in delivery is the steepest from the rabbit eye to the porcine eye. These differences may be due to differences in pigmentation in addition to eye size. Prior studies indicate that some of the differences between species may be due to differences in pigment binding of drugs^19^. Alternatively, the longer delay between isolation and use of rabbit eye may have contributed to this difference.

Most interestingly, a single MLR equation could predict drug delivery to a given tissue in all species, when 1/d^2^ term is incorporated along with commonly used physicochemical properties for predicting drug delivery. The MLR equation for retina explained 74% of the variance in data, while the equation for vitreous humor explained 93% of the data variance. Thus, 1/d^2^ parameter is a particularly useful parameter to scale suprachoroidal drug delivery between species. This parameter was used since the time for diffusion is proportional to the square of distance, as per Fick’s second law. Thus, diffusion-based delivery across the eye globe diminishes in a manner proportional to 1/d^2^. Additionally, the mass of drug dosed in the suprachoroidal space is diluted by the surface area of the globe, which is proportional to the square of the eye diameter, further contributing to the 1/d^2^ decline in delivery efficiency. Based on the findings of this study, it is evident that suprachoroidal delivery to the retina and vitreous humor correlates well between species and the differences in the magnitude of delivery can be explained largely by differences in eye diameter. Further, a combination of four or three drug properties can explain majority of the variance in the delivery to the retina and vitreous humor.

A limitation of the current study is that the experiments were conducted ex vivo over a short duration and the delivery in vivo is more complex. Therefore, the trends observed in vitro must be confirmed in vivo. The present study evaluated the static barriers of the eye and did not factor in the dynamic barriers including blood and fluid flow in the eye. One ex vivo study indicated that perfusion of the eye can affect suprachoroidal drug delivery to the tissues in the back of the eye, with the clearance of the drugs by perfusion being greater for a hydrophilic dye, when compared to a lipophilic dye^20^. Thus, in vivo vascular perfusion might limit the delivery of hydrophilic drugs more than lipophilic drugs. Another limitation of this study is that some drugs used in this study were unstable either due to prodrug nature, or for other reasons (difluprednate and flupirtine maleate, with the latter affected by fluorescein dye in the cassette solution). Therefore, the related prodrug-drug pairs, difluprednate, and flupirtine maleate were excluded from the regression analyses. Additionally, triamcinolone hexacetonide was not included due to high variability of the tissue data. Prodrug/drug delivery data indicated that near complete conversion of the prodrugs to the drugs. However, since both drug and prodrug were used for delivery purposes, dissecting individual contribution of drug and prodrug to delivery is not possible. For a study involving tissue isolation like the current study, it is difficult to divide into two equal parts or to extract the entire tissue. Therefore, such variations may have partly introduced errors in measuring the actual concentration in each half of the eye. While rabbit eyes used in this study are non-pigmented, bovine and porcine eyes are pigmented. Another limitation is that rabbit eyes were used the next day, while porcine and bovine eyes were used on the day of isolation.

## Conclusion

Using isolated, intact eyes, we evaluated suprachoroidal delivery of a drug cassette containing a variety of drugs including beta-blockers, non-steroidal anti-inflammatory agents, and corticosteroids. The drug levels were analyzed in the retina and vitreous humor using a validated LC-MS/MS method. The general trends in drug delivery were similar across species, with the differences being largely explained by variations in eye diameters. Specifically, delivery to the target tissues generally decreased with an increase in eye diameter. Multiple linear regression models were developed to explain drug delivery to the retina and vitreous humor for each species. In this study, suprachoroidal retinal delivery generally increased with drug lipophilicity, both across all compounds and within individual drug classes. Vitreous humor delivery decreased with increasing lipophilicity for beta-blockers and NSAIDs but increased for corticosteroids. Finally, by incorporating an eye geometry related parameter in conjunction with drug properties, a single MLR equation could explain the suprachoroidal drug delivery to a given tissue for all species. While ex vivo tissues lack dynamic physiological barriers present in vivo, it may serve as a valuable platform for preliminary screening of multiple drug candidates or their formulations before proceeding to vivo studies.

## Supplementary Information

Below is the link to the electronic supplementary material.

Supplementary Material 1

## Data Availability

The data obtained from these studies are presented within the manuscript.
