# Supplementary material for: Interspecies scaling of suprachoroidal drug delivery using ocular geometry and drug physicochemical properties
Source: Sci Rep. 2026 Jan 16;16:4502. doi: 10.1038/s41598-025-34631-x (PMC12864957; doi:10.1038/s41598-025-34631-x)
Supplement: Supplementary file 1 — Supplementary Material 1 [file 41598_2025_34631_MOESM1_ESM.docx]

**Supplemental Figures**


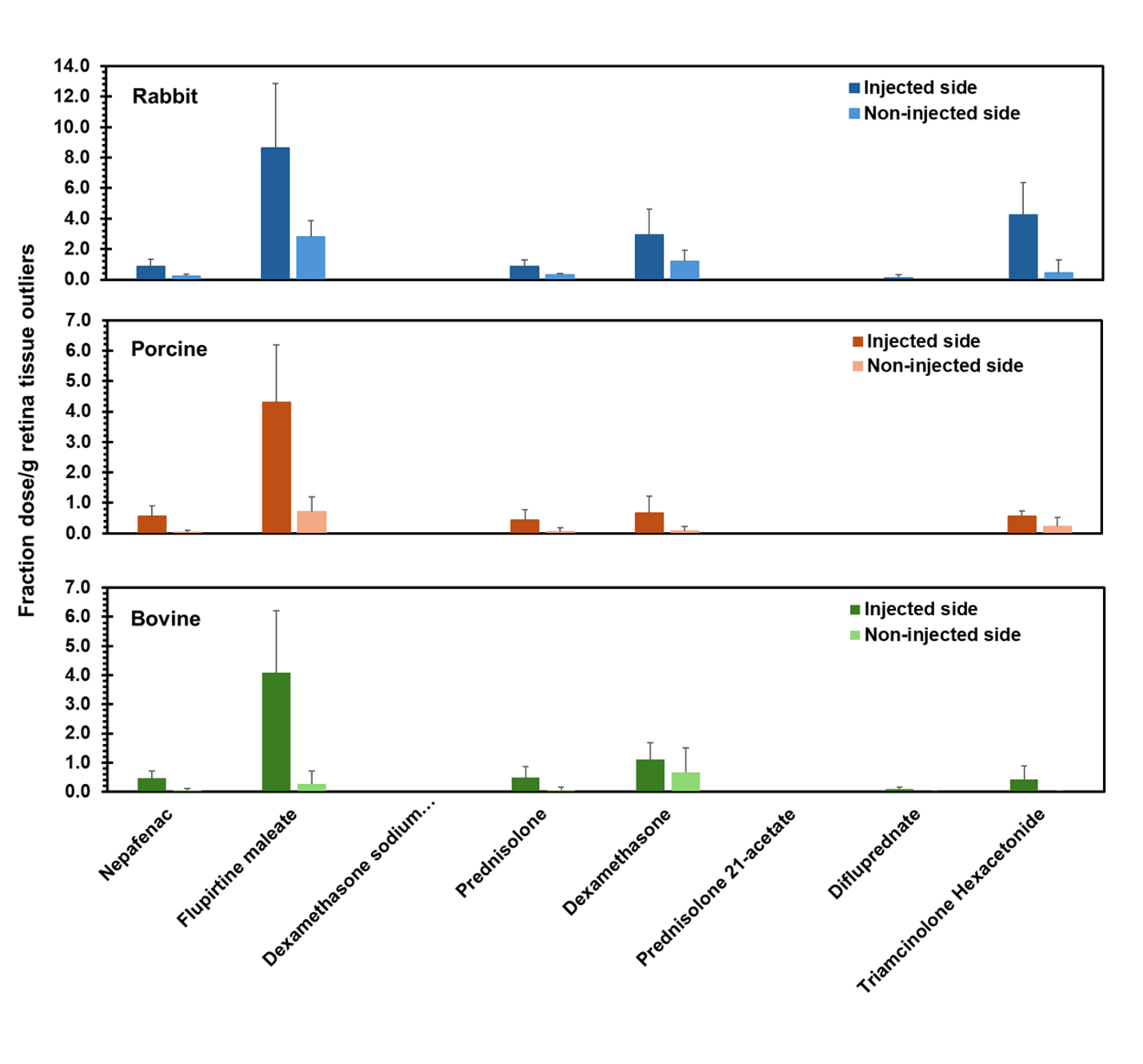


**Supplemental Fig. 1****. Retinal drug delivery to the injected and non-injected side of eyes from three species at 1-hour following suprachoroidal injection.** The data is presented as mean ± STDEV for n=6 eyes, outliers.


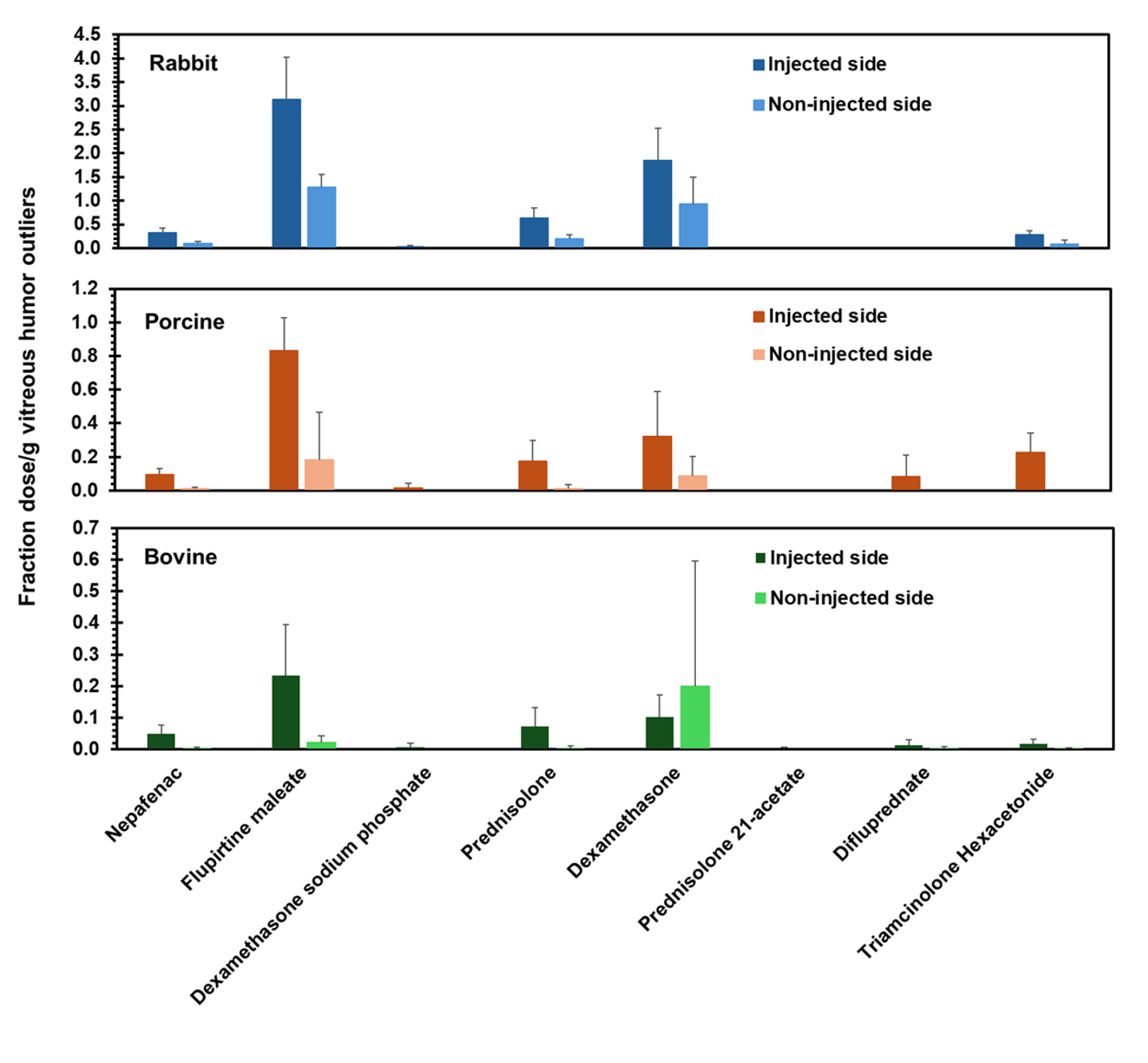


**Supplemental Fig. 2. Vitreous humor drug delivery to the injected and non-injected side of eyes from three species at 1-hour following a suprachoroidal injection.** The data is presented as mean ± STDEV for n=6 eyes, outliers.


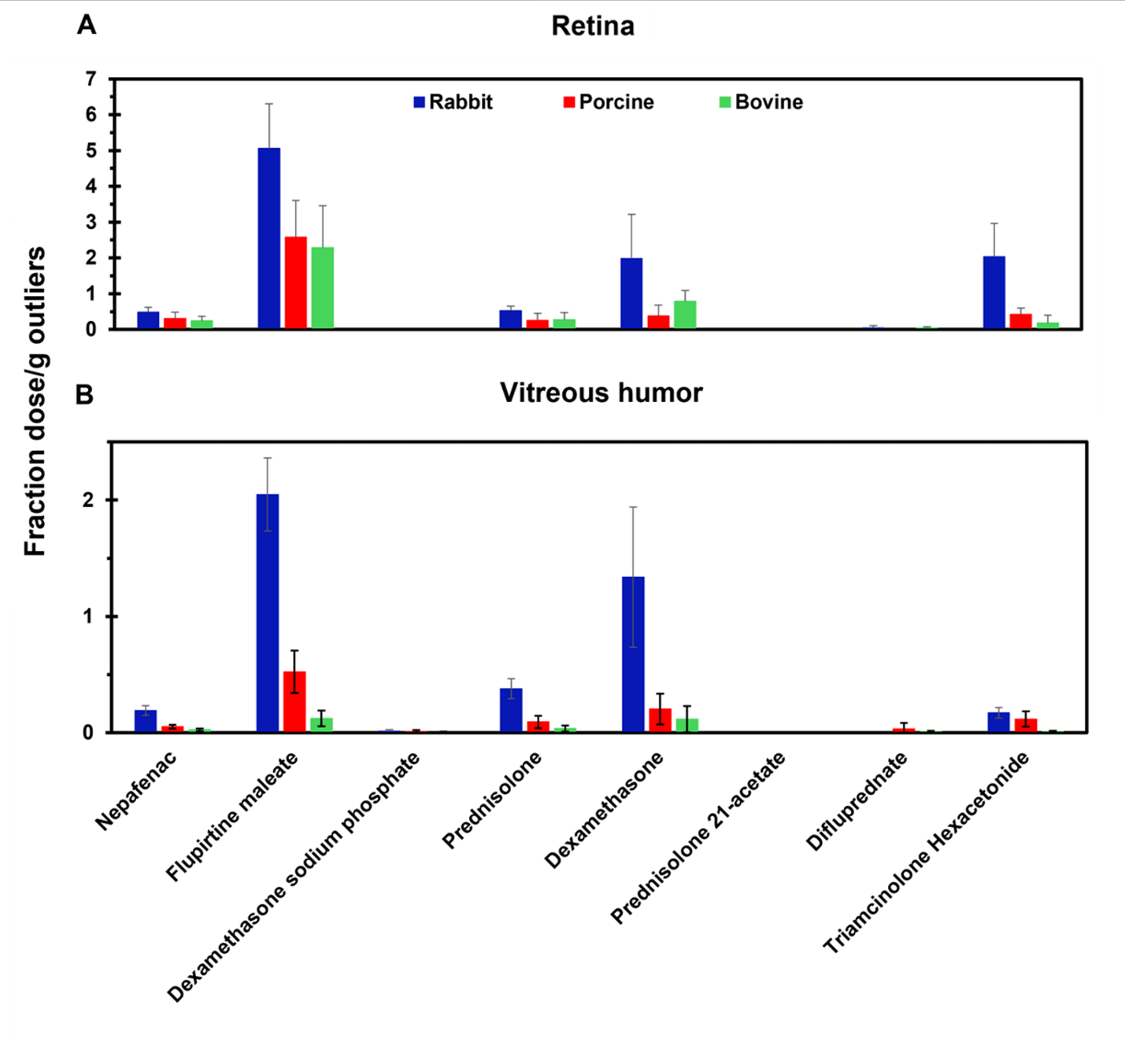


**Supplemental Fig. 3****. Panels A and B show the total retinal and total vitreous humor drug delivery to the injected and non-injected side in the eyes of three species at 1-hour following a suprachoroidal injection.** The data is presented as mean ± STDEV for n=6 eyes, outliers.


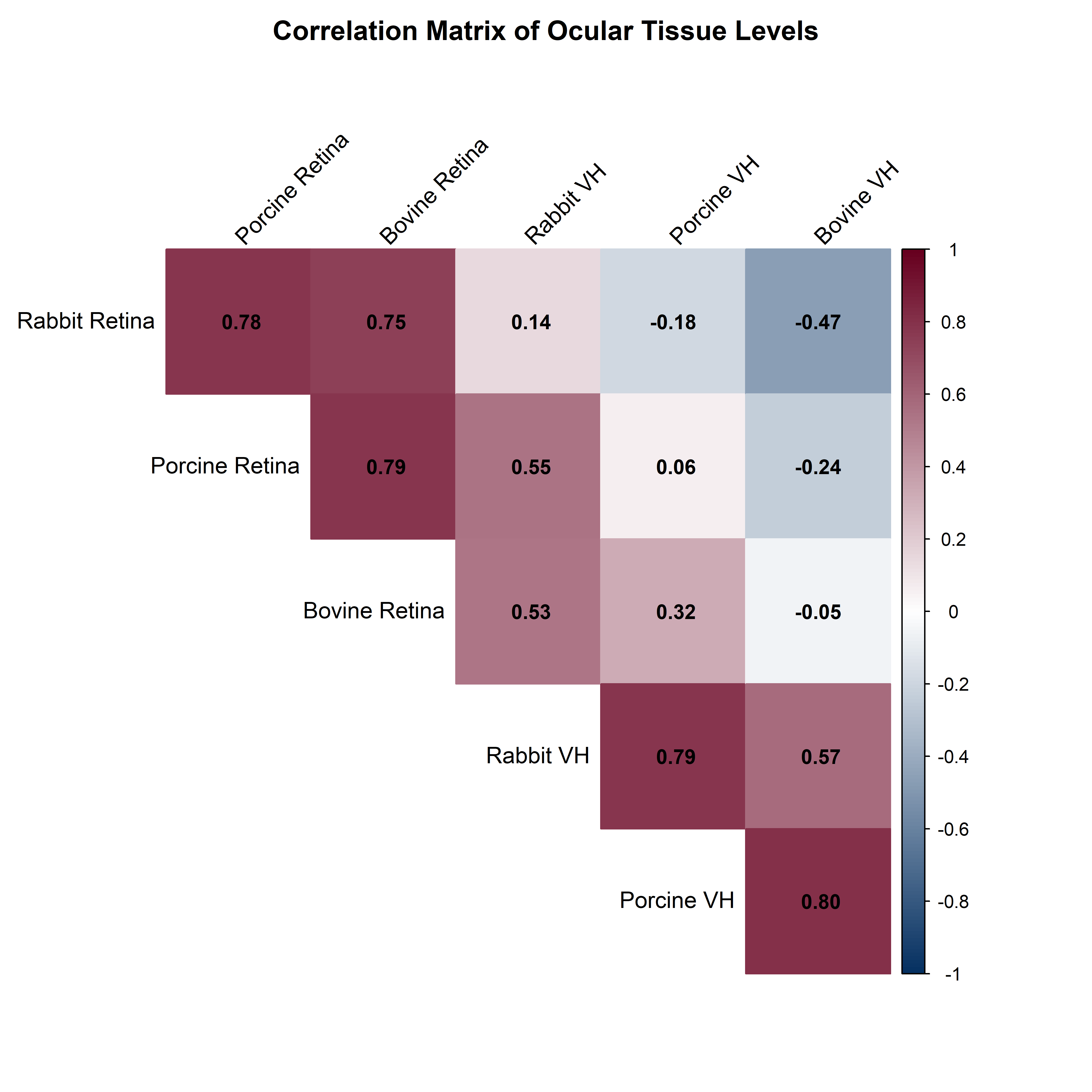
**Supplemental Fig. 4. Pearson’s correlation coefficients for the inter-tissue and inter-species dug concentration relationships following suprachoroidal injection.** Retinal tissue concentrations correlate well between species. Good correlations were also observed between the vitreous humor concentrations of different species. Total drug concentration combining injected and non-injected side was used. Outliers were omitted.


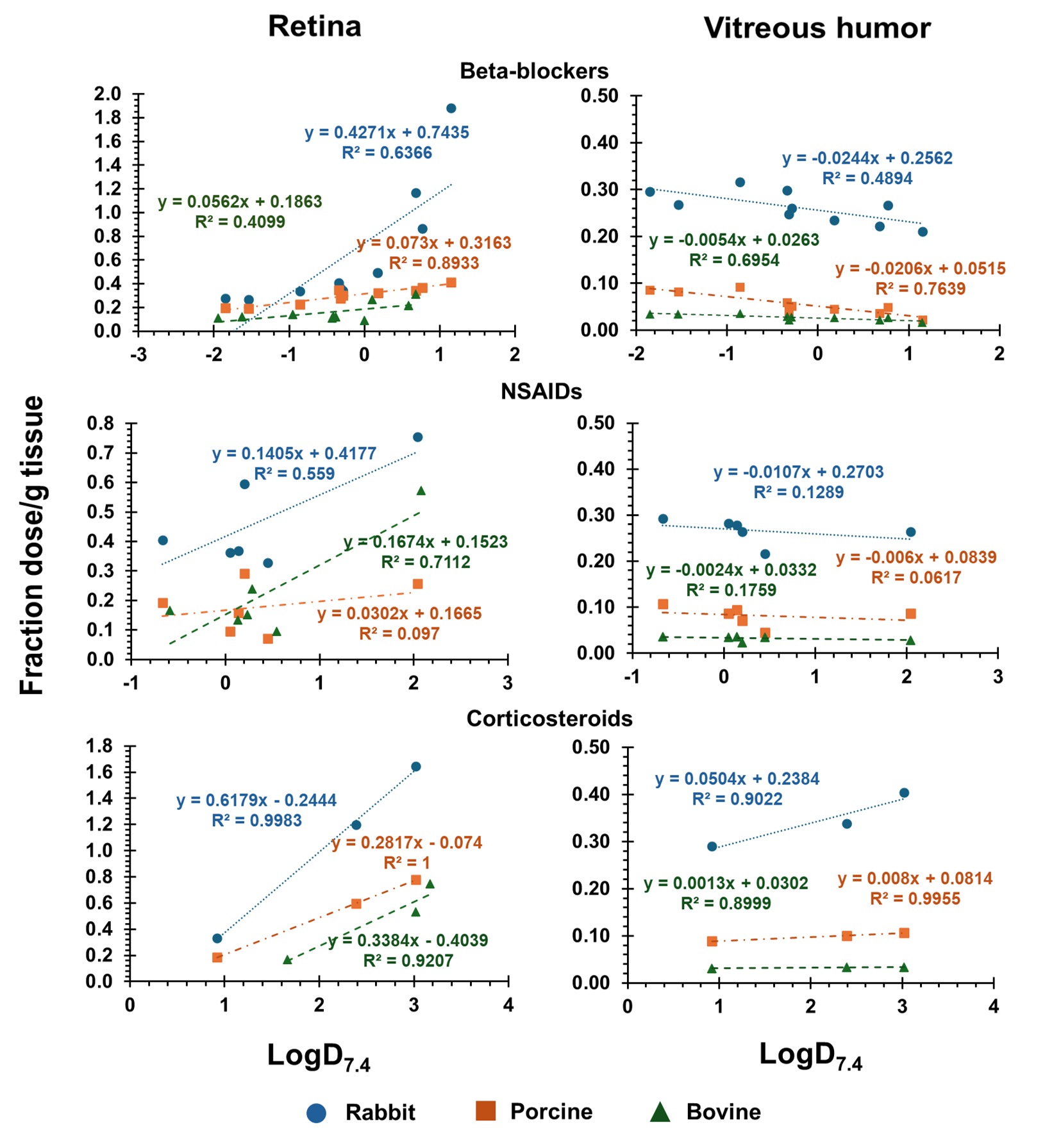


**Supplemental Fig. 5. Correlation of suprachoroidal drug delivery to the retina or vitreous humor with lipophilicity in three species by drug class.** Total drug concentration combining injected and non-injected side was used. Outliers were omitted.
